# Celastrol Inhibits Dopaminergic Neuronal Death of Parkinson’s Disease through Activating Mitophagy

**DOI:** 10.3390/antiox9010037

**Published:** 2019-12-31

**Authors:** Ming-Wei Lin, Chi Chien Lin, Yi-Hung Chen, Han-Bin Yang, Shih-Ya Hung

**Affiliations:** 1 Department of Medical Research, E-Da Hospital/E-Da Cancer Hospital, I-Shou University, Kaohsiung 82445, Taiwan; ta990074@gmail.com; 2 Regenerative Medicine and Cell Therapy Research Center, Kaohsiung Medical University, Kaohsiung 80708, Taiwan; 3 Department of Life Sciences, Institute of Biomedical Science, National Chung Hsing University, Taichung 40249, Taiwan; lincc@dragon.nchu.edu.tw (C.C.L.); alex23206567@yahoo.com.tw (H.-B.Y.); 4 Graduate Institute of Acupuncture Science, China Medical University, Taichung 40402, Taiwan; yihungchen@mail.cmu.edu.tw; 5 Chinese Medicine Research Center, China Medical University, Taichung 40402, Taiwan; 6 Division of Colorectal Surgery, China Medical University Hospital, Taichung 40447, Taiwan

**Keywords:** autophagy, celastrol, dopaminergic neurons, mitophagy, Parkinson’s disease

## Abstract

Parkinson’s disease (PD) is a neurodegenerative disease, which is associated with mitochondrial dysfunction and abnormal protein accumulation. No treatment can stop or slow PD. Autophagy inhibits neuronal death by removing damaged mitochondria and abnormal protein aggregations. Celastrol is a triterpene with antioxidant and anti-inflammatory effects. Up until now, no reports have shown that celastrol improves PD motor symptoms. In this study, we used PD cell and mouse models to evaluate the therapeutic efficacy and mechanism of celastrol. In the substantia nigra, we found lower levels of autophagic activity in patients with sporadic PD as compared to healthy controls. In neurons, celastrol enhances autophagy, autophagosome biogenesis (Beclin 1↑, Ambra1↑, Vps34↑, Atg7↑, Atg12↑, and LC3-II↑), and mitophagy (PINK1↑, DJ-1↑, and LRRK2↓), and these might be associated with MPAK signaling pathways. In the PD cell model, celastrol reduces MPP^+^-induced dopaminergic neuronal death, mitochondrial membrane depolarization, and ATP reduction. In the PD mouse model, celastrol suppresses motor symptoms and neurodegeneration in the substantia nigra and striatum and enhances mitophagy (PINK1↑ and DJ-1↑) in the striatum. Using MPP^+^ to induce mitochondrial damage in neurons, we found celastrol controls mitochondrial quality by sequestering impaired mitochondria into autophagosomes for degradation. This is the first report to show that celastrol exerts neuroprotection in PD by activating mitophagy to degrade impaired mitochondria and further inhibit dopaminergic neuronal apoptosis. Celastrol may help to prevent and treat PD.

## 1. Introduction

Parkinson’s disease (PD) is the second most common neurodegenerative disorder in the world after Alzheimer’s disease (AD), affecting an estimated 7 to 10 million patients globally [1]. The pathological hallmark of PD is a progressive loss of dopaminergic neurons in the substantia nigra pars compacta of the brain, ultimately resulting in severe striatal dopamine deficiency and the development of primary motor symptoms, including resting tremor, bradykinesia, muscle rigidity, and postural instability [2,3]. Many of the genes associated with familial PD also implicate mitochondria during disease pathogenesis such as DJ-1, PINK1, leucine-rich repeat kinase 2 (LRRK2); therefore, PD is described as a mitochondrial disease of aging, as mutations in *DJ-1*, *PINK1*, and *LRRK2* genes are involved in mitophagy that affects mitochondrial quality control in PD [2]. Levodopa has been used for over 50 years to improve motor symptoms, but unfortunately, although drug therapy may initially improve motor symptoms of PD, the benefits frequently wear off over time or become less consistent [4].

Autophagy is a fundamental process that degrades and recycles cellular components (e.g., damaged organelles, abnormal protein aggregates) by enveloping the selected substrate within autophagosomes and fusing them with lysosomes for the substrate digestion by lysosomal hydrolases [5]. The process of autophagy includes autophagy induction, substrate recognition and selection, autophagosome biogenesis (phagophore nucleation/induction, phagophore elongation, substrate binding, and vacuole formation), autophagosome-lysosome fusion, and substrate degradation and recycling [5,6]. Over 30 genes participate in autophagy induction and autophagosome biogenesis [7]. Beclin 1 regulates the autophagic pathway by interacting with several cofactors, including Vps34 (PI3KC3), Vps15, and Ambra1, to form the Beclin 1–hVps34–Vps15 core complex, which is a key element in autophagy induction [8]. During autophagosome biogenesis, the cofactors Atg5, Atg7, Atg16L, Atg10, and Atg12 regulate phagophore formation, while LC3, Atg3, and Atg4B regulate vacuole formation [5,7]. Since autophagy facilitates the reduction of unfolded proteins and dysfunctional mitochondria in neurons, autophagy activity is correlated with disease progression in neurodegenerative disorders such as AD and PD [9].

Mitochondria, double membrane-bound organelles in the cytoplasm of cells, participate in multiple cellular processes, including energy production, calcium homeostasis, metabolic synthesis, and apoptosis [10]. Mitophagy is the selective autophagic degradation of mitochondria [11]. PINK1 is a mitochondrial serine/threonine-protein kinase; loss of PINK1 function alters mitochondrial dynamics and impairs mitochondria, which is associated with the development of PD [12]. DJ-1 is a ubiquitous cytoprotective protein that acts as an antioxidant to protect cells against oxidative stress and maintains mitochondrial health by activating mitophagy [13,14]. PINK1 and DJ-1 can induce mitophagy and thus play a neuroprotective role in neurodegenerative disorders. *LRRK2* mutations are the most common cause of autosomal-dominant PD that can impair depolarization-induced mitophagy; *LRRK2* overexpression induces mitochondrial fragmentation and dysfunction [15,16].

Celastrol, a plant-derived triterpene known as “Thunder of God Vine” in traditional Chinese medicine, has potent antioxidant, anti-inflammatory, antitumor, and neuroprotective activities [17,18]. Celastrol activates autophagy via the ROS/JNK (c-Jun NH2-terminal kinase) signaling pathway in human osteosarcoma cells [18]. Although the mammalian target of the serine/threonine kinase Akt (also known as protein kinase B or PKB), rapamycin (mTOR), and phosphoinositide 3-kinase (PI3K) signaling cascades are considered primary autophagy regulatory pathways and are extensively researched, the MAPK/JNK signal transduction pathway also plays a pivotal role in autophagy [19]. Only two studies have evaluated the efficacy of celastrol in the treatment of PD. The first study shows that celastrol induces heat shock protein 70 in dopaminergic neurons and decreases levels of tumor necrosis factor-alpha and nuclear factor kappa B against 1-methyl 4-phenyl-1,2,3,6-tetrahydropyridine hydrochloride (MPTP)-induced neurotoxicity [20]. The second one shows that celastrol protects SH-SY5Y neuroblastoma cells from rotenone-induced injuries through autophagy induction [21].

Mitochondria were first implicated in PD when it was found that the metabolite 1-methyl-4-phenylpyridinium (MPP^+^) of 1-methyl 4-phenyl-1,2,3,6-tetrahydropyridine hydrochloride (MPTP), a mitochondrial neurotoxin, enters dopaminergic neurons through dopamine transporters and inhibits complex I of the mitochondrial electron transport chain, causing parkinsonism in designer-drug abusers [22]. This dopaminergic specificity of MPP^+^ and MPTP is useful for PD research. MPP^+^ accumulates in mitochondria via an energy-dependent process that interferes with NADH-linked oxidation of pyruvate or glutamate, without affecting the oxidation of succinate [23]. MPP^+^-induced cell death is an autophagic response induced by oxidative stress and mediated by the JNK (activation) and Akt/mTOR (inactivation) signaling pathways [24].

PD is a progressive neurodegenerative disorder; levodopa has been used for over 50 years to improve motor symptoms but not slow down the dopaminergic neuronal death or cure PD. Disease-modifying therapies are urgently needed in PD treatment. Autophagy is a therapeutic approach of PD by removing impaired mitochondria and toxic protein aggregations to prevent neuronal death. Celastrol activates autophagy in human osteosarcoma cells and protects dopaminergic neurons against MPTP-induced neurodegeneration [18,20]. As the neuroprotective effect of celastrol may be multifactorial (e.g., antioxidant, anti-inflammatory, autophagy activation) and PD is a mitochondrial disease of aging, which is associated with oxidative stress, inflammation, and mitophagy. In the present study, we used MPP^+^-induced cell and MPTP-induced mouse models of PD to investigate the neuroprotective mechanisms of celastrol and evaluate celastrol in the regulation of mitophagy.

## 2. Materials and Methods

### 2.1. Microarray Analysis

The GSE8397 microarray gene expression dataset was downloaded from the National Center for Biotechnology Information (NCBI) Gene Expression Omnibus (https://www.ncbi.nlm.nih.gov/geo/query/acc.cgi?acc=GSE8397) and used to analyze substantia nigra samples from neuropathologically confirmed cases of sporadic PD (15 medial and 9 lateral portions) and healthy controls (8 medial and 7 lateral portions) [25]. The clinical and neuropathological characteristics of each case were described previously [25]. Between-group differences were assessed by the Mann–Whitney U test and *p* < 0.05 is considered statistically significant.

### 2.2. Reagents

MPTP and MPP^+^ were purchased from Sigma-Aldrich (St. Louis, MO, USA); celastrol was purchased from Cayman Chemical (Ann Arbor, MI, USA); rapamycin, wortmannin, and bafilomycin A1 were purchased from Enzo Life Sciences, Inc. (Farmingdale, NY, USA).

### 2.3. Cell Culture, MTT, Celastrol Treatment, and the PD Cell Model

The dopaminergic neuronal cell line SH-SY5Y (undifferentiated; American Type Culture Collection; CRL-2266) was maintained in a humidified incubator with 5% CO_2_ at 37 °C in Ham’s F12 Nutrient Mixture/Minimum Essential Medium (Thermo Fisher Scientific, Waltham, MA, USA) supplemented with 10% heat-inactivated fetal bovine serum (HyClone, South Logan, UT, USA), 4500 mg/L glucose, 1% non-essential amino acids (Thermo Fisher Scientific, Waltham, MA, USA) and 1% antibiotic-antimycotic (Thermo Fisher Scientific, Waltham, MA, USA). Cell viability was detected by determining the reduction of 3-(4,5-dimethylthiazole-2yl)-2,5-diphenyltetrazolum bromide (MTT; Thermo Fisher Scientific, Waltham, MA, USA). Cells were seeded at a density of 1.5 × 10^4^ cells in a 96-well plate and exposed to drugs for 24 h, then incubated with 0.5 mg/mL MTT for 2 h. The media was aspirated and 0.1 mL dimethylsulfoxide was added. Absorbance was measured at 570 nm using a microplate reader (Bio-Tek Synergy HT; BioTek^®^ Instruments, Inc., Winooski, VT, USA). According to the different experimental settings, SH-SY5Y cells were treated with 0.1–3 μM celastrol for 2–24 h, which is indicated in figure legends. For the PD cell model, SH-SY5Y cells were treated with 1 mM MPP^+^ for 24 h to induced about 50% neuronal death.

### 2.4. Western Blot

Substantia nigra and striatal tissues were dissected by using a mouse brain slicer matrix with 1.0 mm coronal section slice intervals according to our previous study [3]. Western blot analysis determined protein expression levels from cells, substantia nigra, and striatal tissues. Samples obtained from cells or tissues were homogenized in radioimmunoprecipitation assay buffer containing 1% protease inhibitor cocktail (Hycell, Taipei, Taiwan) and 1% phosphatase inhibitor cocktail (Hycell, Taipei, Taiwan). The protein concentration was determined by a BCA protein assay kit (Thermo Fisher Scientific, Waltham, MA, USA). Protein at 20–40 μg was separated by SDS-PAGE using a 10–15% resolving gel under reducing conditions and electrotransferred onto Immun-Blot^®^ PVDF Membrane (Bio-Rad, Hercules, CA, USA). After being blocked with 5% nonfat milk in 0.5% Tween 20 in 20 mM Tris and 137 mM NaCl for 1 h at room temperature, the membranes were incubated overnight at 4 °C with anti-pp38 (Elabscience, Houston, TX, USA; E-AB-21027), anti-p38 (Cell Signaling Technology, Danvers, MA, USA; 9212), anti-pERK1/2 (Cell Signaling Technology, Danvers, MA, USA; 4695S), anti-ERK1/2 (Cell Signaling Technology, Danvers, MA, USA; 4695S), anti-pAkt1/2/3 (GeneTex, Irvine, CA, USA; GTX128414), anti-Akt1/2/3 (Cell Signaling Technology, Danvers, MA, USA; 4691), anti-pNFκBP65 (GeneTex, Irvine, CA, USA; GTX50254), anti-pJNK1/2/3 (GeneTex, Irvine, CA, USA; GTX50868), anti-LC3 (GeneTex, Irvine, CA, USA; GTX127375), anti-p62 (GeneTex, Irvine, CA, USA; GTX100685 or Elabscience, Houston, TX, USA; E-AB-63539), anti-tyrosine hydroxylase (Elabscience, Houston, TX, USA; E-AB-33093), anti-Beclin 1 (Cell Signaling Technology, Danvers, MA, USA; 3495), anti-Vps34 (Novus, St. Charles, MO, USA; NB110-87320), anti-Atg7 (Cell Signaling Technology, Danvers, MA, USA; 8558), anti-β-actin (ProteinTech, Rosemont, IL, USA; 60008), anti-GAPDH (ProteinTech, Rosemont, IL, USA; 60004), anti-PINK1 (Cell Signaling Technology, Danvers, MA, USA; 6946), anti-DJ-1 (Cell Signaling Technology, Danvers, MA, USA; 5933), anti-LRRK2 (Cell Signaling Technology, Danvers, MA, USA; 13046), anti-Ub (Santa Cruz Biotechnology, Dallas, TX, USA; sc-8017), or anti-EF-Tu (Santa Cruz Biotechnology, Dallas, TX, USA; sc-393924) primary antibodies diluted in TBS-T at 4 °C overnight. The blots were then incubated for 1 h at room temperature with an HRP-conjugated secondary antibody (1:20,000; Santa Cruz Biotechnology, Dallas, TX, USA; sc-2004 or sc-2005). Protein bands were detected using the ECL Western blot substrate kit (Bio-Rad, Hercules, CA, USA) and ImageQuant LAS 4000 mini biomolecular imager (GE Healthcare Life Sciences, Uppsala, Sweden) and then estimated using Fusion software (VilBER, Collégien, France).

### 2.5. Mitochondrial Membrane Potential Analysis and ATP Production Assay

Changes in mitochondrial membrane potential induced by MPP^+^ were measured with JC-10^TM^ dye (ATT Bioquest, Sunnyvale, CA, USA) using a fluorescence microplate reader (Bio-Tek Synergy HT, BioTek^®^ Instruments, Inc., Winooski, VT, USA) according to the manufacturer’s protocol. ATP production of cells was measured by an ATP colorimetric assay kit (BioVision, San Francisco, CA, USA) as described previously [26].

### 2.6. The PD Mouse Model

Male C57BL6 mice (aged 4–8 weeks; BioLasco Taiwan Co., Ltd., Taipei, Taiwan) were used. They were housed in our animal facility under a 12 h light/dark cycle with food and water available *ad libitum* for at least 4 days before the experiments. The animal use protocol (China Medical University protocol number: 2016-198) was reviewed and approved by the Institutional Animal Care and Use Committee (IACUC) of China Medical University according to the principles of the 3Rs (replacement, reduction, and refinement). MPTP and celastrol dosages for mice studies were according to the previous studies [3,20]. Mice received intraperitoneal (i.p.) injections of MPTP (10 mg/kg/day for 3 days) 24 h after the last celastrol injection (3 mg/kg/day for 3 days; Figure 3A).

### 2.7. Cylinder Task and Accelerated Rotarod Test

Cylinder task was used to assess spontaneous forelimb use, while the accelerated Rotarod test assessed motor coordination and balance in the MPTP mouse model of PD [27]. The cylinder task was performed on the last study day (Day 11). Each mouse was placed for 5 min into a transparent cylinder measuring 11.5 cm in diameter and 25 cm in height and videotaped during the test [28]. The number of forepaw contacts to the cylinder wall was counted manually. Before the celastrol and MPTP injections (Day 1), rotarod testing recorded the length of time a mouse remained on a rotating rod (Ugo Basile S.R.L., Monvalle, Italy) with auto acceleration from 0 rotation per minute (rpm) to 40 rpm in 5 min (every 10 s plus 5 rpm), to obtain latency-to-fall baseline values. On the last day (Day 11), rotarod testing was performed again to study the effect of celastrol in the MPTP-induced PD mouse models.

### 2.8. Immunohistochemistry Staining of Tyrosine Hydroxylase

Brain tissue sections (30 μm thickness) were pretreated with 3% hydrogen peroxide to eliminate endogenous peroxidase activity, blocked/permeabilized in 10% goat serum/0.1% Triton X-100 in phosphate-buffered saline (PBS) before added the anti-tyrosine hydroxylase antibody (R&D Systems, Inc., Minneapolis, MN, USA; MAB7566), and then visualized with 0.01% hydrogen peroxide and 0.05% 3,3′-diaminobenzidine (Sigma-Aldrich, St. Louis, MO, USA) as described previously [3]. Immunohistochemistry images were quantified using ImageJ software (National Institutes of Health, Bethesda, MD, USA) according to Vinet et al. (2018) [29]

### 2.9. RNA Extraction, Reverse-Transcription, and Real-Time Quantitative PCR

Total RNA for gene expression was extracted from cells or tissues according to the NucleoSpin^®^ RNA protocol (MACHEREY-NAGEL GmbH & Co. KG, Düren, Germany). Single-strand cDNA was synthesized using a high-capacity cDNA Reverse Transcription kit (Thermo Fisher Scientific, Waltham, MA, USA). Real-time quantitative PCR was performed to analyze gene expression using the StepOne Plus real-time PCR system (Thermo Fisher Scientific, Waltham, MA, USA). Table 1 shows primer and probe sequences for each reaction of real-time quantitative PCR. Thermal cycling conditions were 95 °C for 10 min, 40 cycles at 95 °C for 10 s, 55 °C for 30 s, then 72 °C for 10 s. RNA expression levels were initially normalized with β-actin mRNA and then expressed as control relative to overexpression by the ΔΔCt method as described previously [30].

### 2.10. Immunofluorescence Double Staining, Colocalization Analysis, and Mitochondrial Isolation

For labeling of the autophagosomes and mitochondria, SH-SY5Y cells were plated at a density of 1.8 × 10^5^ cells/well in 24-well plates and seeded in 500 μL medium overnight. After being treated with 50 nM bafilomycin for 30 min and then celastrol for 4 h, cells were fixed, blocked/permeabilized with 10% bovine serum albumin/0.5% Triton X-100, stained with antibodies against LC3 (1:500; GeneTex, Irvine, CA, USA; GTX127375) and EF-Tu (1:500; Santa Cruz Biotechnology, Dallas, TX, USA; sc-393924), and then with Alexa-488-conjugated goat anti-rabbit secondary antibody (1:1000, Thermo Fisher Scientific, St. Louis, MO, USA, A-11008) and Alexa-543-conjugated goat anti-mouse secondary antibody (1:1000, Thermo Fisher Scientific, St. Louis, MO, USA, A-11003) as described previously [31]. Here, we used rabbit and mouse IgG isotype controls (GeneTex, Irvine, CA, USA; GTX35035 and GTX35009) to replace anti-LC3B and anti-EF-Tu antibodies as negative controls. Confocal images were obtained using excitation wavelengths of 488 nm (for Alexa-488) or 543 nm (for Alexa-543) by Leica confocal microscope (TCS SP8, Leica, Wetzlar, Germany). The colocalization percentage of LC3 and EF-Tu in each cell was analysis by Leica Application Suite X software (LAS X, Leica, Wetzlar, Germany). Mitochondria were isolated from the SH-SY5Y cells using a Mitochondria Isolation Kit (Thermo Fisher Scientific, St. Louis, MO, USA) according to the manufacturer’s protocol.

### 2.11. Statistics

Results are expressed as the mean ± standard error of mean (SEM). Except for microarray data, all data were analyzed with the Kruskal–Wallis test followed by Dunn’s multiple comparison post hoc test to determine the between-group statistical significance by GraphPad Prism 5 (GraphPad Software, San Diego, CA, USA). The difference is considered significant when *p* < 0.05.

## 3. Results

### 3.1. PD Patients Have Lower Autophagic Activity in the Substantia Nigra as Compared to Healthy Controls

**Figure 1:**
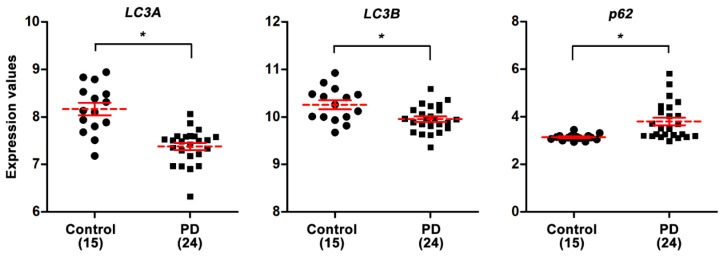
Lowered autophagic activity in the substantia nigra of the midbrain in patients with Parkinson’s disease (PD). Gene expression data (GSE8397) from 24 patients with sporadic PD (15 medial and 9 lateral substantia nigra samples) and 15 normal healthy control (8 medial and 7 lateral substantia nigra samples) were obtained from the National Center for Biotechnology Information (NCBI) Gene Expression Omnibus (GEO) repository. Dot plots with mean + SEM for *LC3A*, *LC3B*, and *p62* mRNA expressions were created from the gene expression datasets. The analysis reveals decreased *LC3A* and *LC3B* mRNA expressions, and increased *p62* mRNA expression, in PD as compared to controls. Comparisons of mean + SEM between the control and PD groups were analyzed using the Partek Genomics Suite software. Statistical significance of between-group differences was analyzed using the Mann–Whitney U test. * *p* < 0.05.

Microtubule-associated protein 1 light chain 3 (LC3) and p62 (also known as sequestosome 1) are widely accepted as markers of autophagosome formation and autophagic flux/substrate degradation, respectively. Levels of mRNA expression relating to *LC3A*, *LC3B*, and *p62* from the medial and lateral portions of the substantia nigra revealed significantly lower autophagic activity among the 24 patients with sporadic PD (*LC3A*↓, *LC3B*↓, and *p62*↑) as compared to that in 15 healthy controls (Figure 1; *p* < 0.05).

### 3.2. Celastrol Activates Autophagy and Inhibits MPP^+^-Induced Neurotoxicity in SH-SY5Y

**Figure 2:**
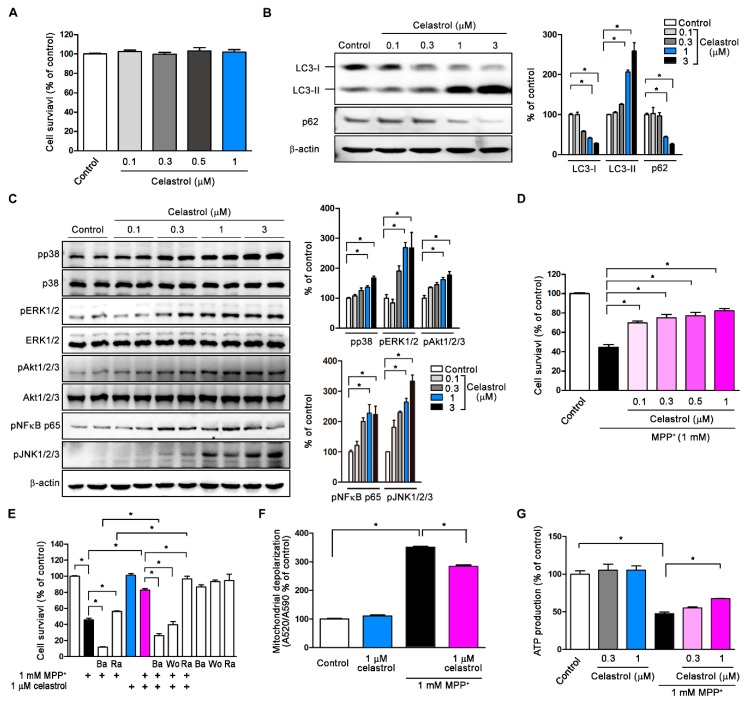
Celastrol activates autophagy and inhibits MPP^+^-induced neurotoxicity in dopaminergic neurons. The dopaminergic cell line SH-SY5Y was treated with celastrol (0.1–3 μM) for 2 to 24 h. (**A**) MTT assay results show that celastrol did not affect cell viability in 24 h. (**B**) Western blot data show that celastrol treatment for 4 h dose-dependently increased autophagosome formation (LC3-I↓ and LC3-II↑) and enhanced autophagic flux/substrate degradation (p62↓) as compared to control. (**C**) Celastrol dose-dependently enhanced p38, ERK1/2, Akt1/2/3, NFκBp65, and JNK1/2/3 phosphorylation 2 h after treatment. (**D**) 1-methyl-4-phenylpyridinium (MPP^+^; 1 mM) but not celastrol treatment for 24 h caused about 50% neuronal apoptosis induced by mitochondrial dysfunction. Celastrol cotreatment dose-dependently suppressed MPP^+^-induced neurotoxicity as compared to MPP^+^. (**E**) Pretreatment with the autophagy inhibitor Ba (100 nM bafilomycin A1) for 30 min before MPP^+^ treatment for 24 h enhanced MPP^+^-induced neuronal death but pretreatment with the autophagy activator Ra (200 nM rapamycin) reversed it. Pretreatment of cells with autophagy inhibitors Ba or Wo (20 nM wortmannin) antagonized the neuroprotection of celastrol against MPP^+^ as compared to MPP^+^+celastrol; pretreatment with the autophagy activator Ra reversed it. Celastrol, Ba, Wo, and Ra did not affect cell viability as compared to control. (**F**) MPP^+^ but not celastrol induced mitochondrial depolarization after treatment for 24 h. Celastrol (1 μM) cotreatment with MPP^+^ suppressed mitochondrial depolarization as compared to MPP^+^. (**G**) MPP^+^ but not celastrol treatment decreased the ATP production after treatment for 24 h. Celastrol (1 μM) cotreatment dose-dependently reversed the ATP production as compared to MPP^+^. Data are given as the mean ± SEM (*n* = 3–8). *p*-value was determined using the Kruskal–Wallis test followed by Dunn’s multiple comparison post hoc test. * *p* < 0.05 compared to control.

We initially treated SH-SY5Y cells with 0.1–1 μM celastrol for 24 h and found that cell viability was not affected (Figure 2A). Western blot results revealed that celastrol dose-dependently increased autophagosome formation by enhancing the conversion of soluble LC3-I to lipid-bound LC3-II and autophagic flux/substrate degradation (p62↓) at 4 h after 0.1–3 μM celastrol treatment (Figure 2B). MAPK (mitogen-activated protein kinase) subfamilies are signaling transduction pathways, some of which regulate autophagy or mitophagy such as MAPK/Akt, MAPK/ERK, and MAPK/JNK [32,33]. After treatment for 2 h, celastrol dose-dependently enhanced p38, ERK1/2, Akt1/2/3, NFκBp65, and JNK1/2/3 phosphorylation (pp38↑, pERK1/2↑, pAkt1/2/3↑, pNFκBp65↑, pJNK1/2/3↑; Figure 2C). Application of MPP^+^ (1 mM) for 24 h induced significant cell death (about 50%) as compared to control; celastrol cotreatment dose-dependently suppressed MPP^+^-induced neuronal death as compared to MPP^+^ alone (Figure 2D). Pretreatment with the autophagy inhibitor Ba (100 nM bafilomycin A1) for 30 min before MPP^+^ treatment for 24 h enhanced MPP^+^-induced cell death; pretreatment with the autophagy activator Ra (200 nM rapamycin) reversed it as compared to MPP^+^ alone (Figure 2E). Indicating autophagy activation inhibits MPP^+^-induced neuronal death. Pretreatment with autophagy inhibitors Ba or Wo (20 nM wortmannin) for 30 min before MPP^+^ treatment for 24 h inhibited the neuroprotection of celastrol against MPP^+^ as compared to MPP^+^+celastrol; pretreatment with the autophagy activator Ra reversed it (Figure 2E). Indicating celastrol actives autophagy to inhibit MPP^+^-induced neuronal death. After treating the cells with 1 mM MPP^+^ for 24 h, JC-10 assay and ATP production results show that MPP^+^ increased the depolarization of mitochondrial membrane potential and reduced ATP production (Figure 2F,G), whereas this phenomenon was antagonized by celastrol (1 μM) cotreatment (Figure 2F,G). These data indicate that (1) celastrol enhances autophagosome formation and autophagy flux/substrate degradation that might be associated with MAPK/p38, MAPK/ERK, MAPK/Akt, or MAPK/JNK signaling pathways; (2) autophagy and mitophagy are involved in celastrol against MPP^+^ neurotoxicity in dopaminergic neurons.

### 3.3. Celastrol Improves Motor Symptoms and Inhibits Dopaminergic Neuronal Degeneration in the Substantia Nigra and Striatum of the PD Mouse Model

**Figure 3:**
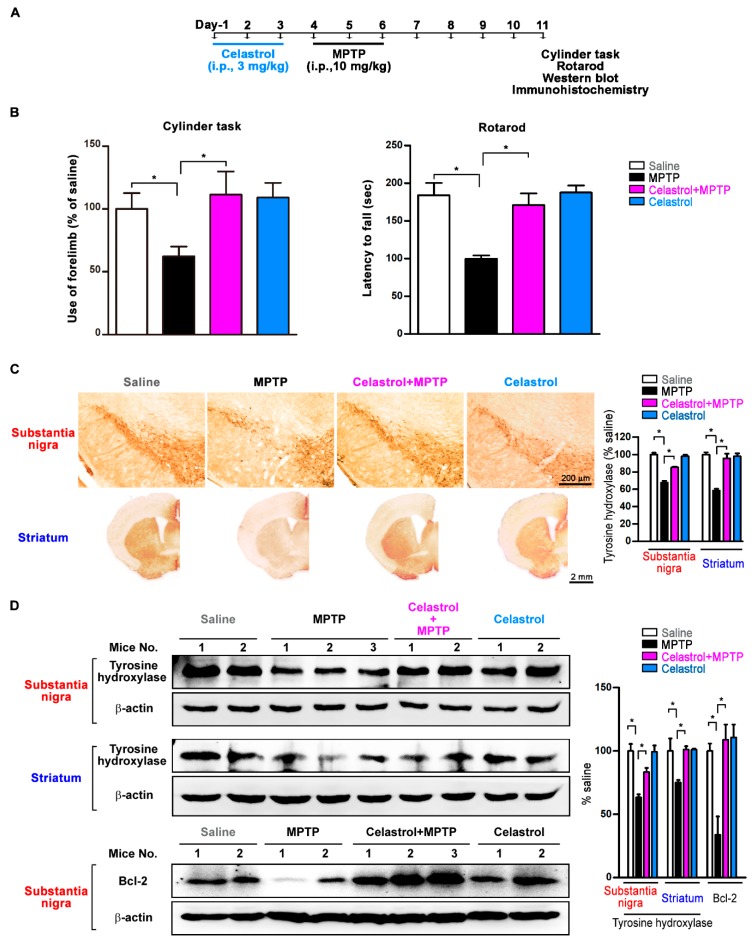
Celastrol improves motor symptoms and inhibits dopaminergic neurodegeneration in the 1-methyl 4-phenyl-1,2,3,6-tetrahydropyridine hydrochloride (MPTP) mouse model of PD. Systemic MPTP injections (i.p., 10 mg/kg/day for 3 days) induced substantia nigra dopaminergic neuronal loss and nerve terminal degeneration in the striatum on both sides of the nigrostriatal dopaminergic pathway. (**A**) The experimental protocol using celastrol to treat MPTP-induced PD mouse models. (**B**) On the last study day (Day 11), cylinder and rotarod results show that MPTP but not celastrol treatment reduced forelimb usage and the latency to fall as compared to saline control. Celastrol cotreatment with MPP^+^ significantly increased forelimb usage and the latency to fall on Day 11 as compared to MPTP. (**C**) Immunohistochemistry staining of tyrosine hydroxylase data indicates that MPTP but not celastrol induced neurodegeneration in the substantia nigra and striatum as compared to saline control. In the celastrol+MPTP group, celastrol exerted neuroprotection in substantia nigra and striatum as compared to MPTP. (**D**) Western blot results show that MPTP but not celastrol induced neurodegeneration (tyrosine hydroxylase↓ and Bcl-2↓) in the substantia nigra and striatum as compared to saline control. In the celastrol+MPTP group, celastrol reversed it (tyrosine hydroxylase↑ and Bcl-2↑) as compared to MPTP. These data indicate that celastrol produces motor improvements and is neuroprotective in the MPTP mouse model of PD. Data are given as the mean ± SEM (*n* = 5–10 in each group). *p*-value was determined using the Kruskal–Wallis test followed by Dunn’s multiple comparison post hoc test. * *p* < 0.05 compared to saline control.

Systemic MPTP injections (10 mg/kg/day for 3 days, i.p.) induced substantia nigra dopaminergic neuronal death and nerve terminal degeneration in the striatum in both sides of the nigrostriatal dopaminergic pathway [3]. Figure 3A depicts the experimental protocol using celastrol to treat MPTP-induced PD mouse models. On the last study day (Day 11), results of cylinder task and accelerated rotarod test show that MPTP but not celastrol (3 mg/kg/day for 3 days, i.p.) treatment reduced forelimb usage and latency to fall as compared to saline-treated controls (Figure 3B). On Day 11, celastrol cotreatment with MPTP significantly increased forelimb usage and the latency to fall off the rotarod as compared to MPTP group (Figure 3B). Tyrosine hydroxylase is a marker for dopaminergic neurons. Immunohistochemistry staining results of tyrosine hydroxylase show that MPTP but not celastrol induced dopaminergic neuronal death in the substantia nigra and terminal degeneration in the striatum as compared to saline control (Figure 3C). In the celastrol+MPTP group, celastrol was neuroprotective against MPTP-induced dopaminergic neuronal death in the substantia nigra and nerve terminal degeneration in the striatum as compared to MPTP group (Figure 3C). Similarly, Western blot results show that MPTP but not celastrol decreased tyrosine hydroxylase expression in the substantia nigra and striatum as compared to saline control (Figure 3D). In the celsatrol+MPTP group, celastrol protected against MPTP-induced neurodegeneration in the substantia nigra and striatum (tyrosine hydroxylase↑) as compared to MPTP (Figure 3D). Results of the anti-apoptotic protein Bcl-2 show that MPTP but not celastrol treatment reduced Bcl-2 expression in the substantia nigra as compared to saline control (Figure 3D). In the celsatrol+MPTP group, celastrol reversed it as compared to MPTP (Figure 3D). These data indicate that celastrol improves motor symptoms and exerts neuroprotective effects in the MPTP mouse model of PD.

### 3.4. Celastrol Regulates Autophagy and Mitophagy-Related Gene Expression

**Figure 4:**
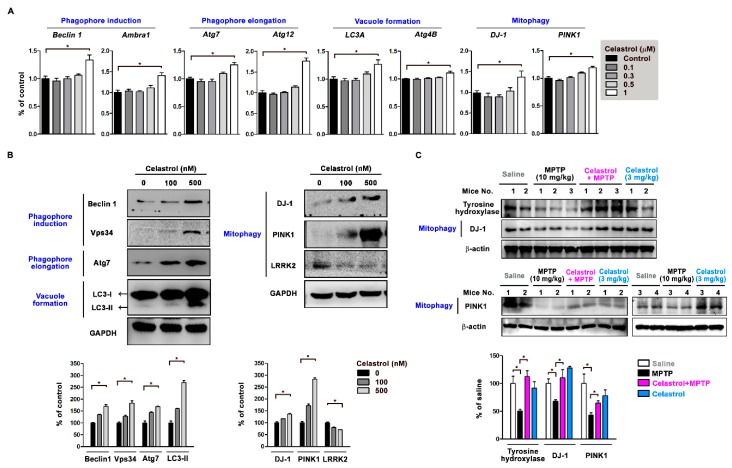
Celastrol regulates autophagy- and mitophagy-related gene expressions in neurons. SH-SY5Y cells treated with 0.1–1 μM celastrol for 4 h. (**A**) Real-time quantitative PCR results show that 1 μM celastrol treatment for 4 h enhanced mRNA expressions of phagophore induction genes *Beclin 1* and *Ambra1*, phagophore elongation genes *Atg7* and *Atg12*, and vacuole formation genes *LC3A* and *Atg4B*. (**B**) Western blot results of SH-SY5Y treated with 100–500 nM celastrol show celastrol (500 nM) increased in protein expressions of Beclin 1 and Vps34 (phagophore induction), Atg7 (phagophore elongation), LC3-II (vacuole formation), DJ-1, and PINK1 (mitophagy). Celastrol (500 nM) suppressed LRRK2 expression. (**C**) MPTP (10 mg/kg/day for 3 days, i.p.) but not celastrol (3 mg/kg/day for 3 days, i.p.) caused dopaminergic nerve terminal degeneration (tyrosine hydroxylase↓) and mitophagy inactivation (DJ-1↓ and PINK1↓) in the striatum of mice as compared to saline control. Celastrol cotreatment with MPTP reversed it as compared to MPTP. Data are given as the mean ± SEM (*n* = 3–5). *p*-value was determined using the Kruskal–Wallis test followed by Dunn’s multiple comparison *post hoc* test. * *p* < 0.05 compared with controls.

Autophagosome biogenesis contains several steps, including phagophore induction, phagophore elongation, and vacuole formation [5]. Beclin 1 is a critical regulator in the initiation of the autophagic process, which involves in phagophore induction [8]. Beclin 1 interacts with Class III phosphatidylinositol 3-kinase (PI3KC3)/VPS34, Ambra1, Vps15, and Atg14L, leading to autophagy activation [8]. The conjugation of LC3-I to PE (LC3-II) is mediated by the E1- and E2-like enzymes Atg7 and Atg3, respectively [5]. Results of real-time quantitative PCR show that SH-SY5Y cells treated with 1 μM celastrol (in the concentration range of 0.1–1 μM) for 4 h enhanced mRNA expressions of phagophore induction genes *Beclin 1* and *Ambra1*, phagophore elongation genes *Atg7* and *Atg12*, and vacuole formation genes *LC3A* and *Atg4B* (Figure 4A). PINK1 and DJ-1 regulate mitophagy [34]. After SH-SY5Y treated with 0.1–1 μM celastrol for 4 h, 1 μM celastrol increased *PINK1* and *DJ-1* mRNA expressions (Figure 4A). Western blot results of SH-SY5Y cells treated with 100–500 nM celastrol reveal that celastrol (500 nM) increased in protein expressions of Beclin 1 and Vps34 (phagophore induction↑), Atg7 (phagophore elongation↑), LC3-II (vacuole formation↑), and DJ-1 and PINK1 (mitophagy↑) (Figure 4B). Also, we found that celastrol (500 nM) suppressed LRRK2 expression, which is associated with mitochondrial fragmentation and mitophagy inactivation (Figure 4B). In mice, we found that MPTP (10 mg/kg/day for 3 days, i.p.) but not celastrol (3 mg/kg/day for 3 days, i.p.) caused dopaminergic nerve terminal degeneration (tyrosine hydroxylase↓) and reduced DJ-1 and PINK1 expressions in the striatum as compared to saline control, while celastrol co-treatment with MPTP reversed tyrosine hydroxylase, PINK1, and DJ-1 protein expressions (Figure 4C). These data indicate that celastrol modulates the process of autophagosome biogenesis and mitophagy to enhance the clearance of impaired mitochondria as quality control and further inhibit neuronal apoptosis.

### 3.5. Celastrol Facilitates Autophagosome Sequestration of Impaired Mitochondria for Mitophagy

**Figure 5:**
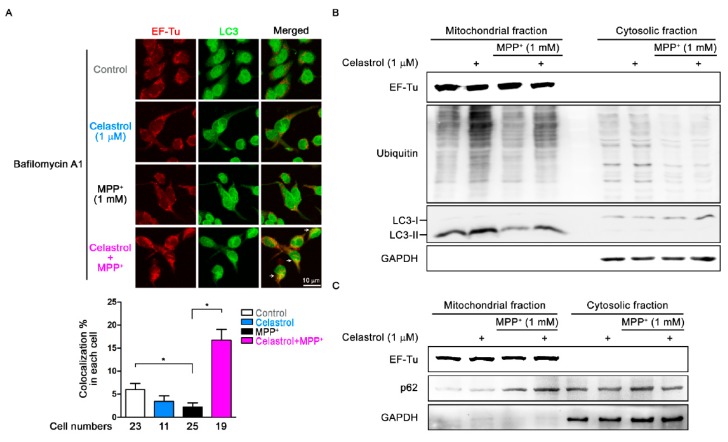
Celastrol enhances the clearance of damaged mitochondria via activating mitophagy. This study used 1 mM MPP^+^ to induce mitochondrial dysfunction and 100 nM bafilomycin A1 pretreatment for 30 min to inhibit autophagosomes fusion with lysosomes in SH-SY5Y cells. (**A**) Images of immunodouble staining obtained from confocal microscopy show that MPP^+^ but not celastrol treatment reduced dysfunctional mitochondria co-localized with autophagosomes as compared to control. In the celastrol + MPP^+^ group, celastrol enhanced MPP^+^-induced dysfunctional mitochondria co-localized with autophagosomes as compared to MPP^+^ alone. Here, we used anti-EF-Tu and anti-LC3 antibodies to stain mitochondria and autophagosomes, respectively. The colocalization of EF-Tu and LC3 was indicated by arrows. Summarized results of the percentage of colocalization in each cell are given as the mean ± SEM (*n* = 11–25). *p*-value was determined using the Kruskal–Wallis test followed by Dunn’s multiple comparison post hoc test. * *p* < 0.05 as compared to control. (**B**) Western blot results of mitochondrial fractions and cytosolic fractions from cells show that celastrol but not MPP^+^ enhanced ubiquitinated and LC3-II bound mitochondria as compared to control in mitochondria fractions; celastrol cotreatment with MPP^+^ enhanced it as compared to MPP^+^ alone. (**C**) MPP^+^ but not celastrol treatment increased p62 conjugation in the mitochondrial fraction as compared to control; celastrol cotreatment with MPP^+^ enhanced it as compared to MPP^+^. Here, we used EF-Tu and GAPDH to identify the purity of mitochondrial and cytosolic fractions, respectively.

In Figure 2F,G, we found MPP^+^ but not celastrol increased the depolarization of mitochondrial membrane potential and inhibited the ATP production in cells, suggesting MPP^+^ induces mitochondrial dysfunction and celastrol (1 μM) reversed it. We then analyzed the effect of celastrol in the clearance of damaged mitochondria. SH-SY5Y cells were pretreated with 100 nM bafilomycin A1 for 30 min to inhibit lysosomal degradation of impaired mitochondria after autophagosome-lysosome fusion and then added 1 mM MPP^+^ for 4 h to induce mitochondrial damage. We used anti-EF-Tu (EF-Tu is the translation elongation factor of mitochondria) and anti-LC3 antibodies to stain mitochondria and autophagosomes, respectively. Immunodouble staining images obtained from confocal microscopy reveal that the percentage of colocalization of mitochondria and autophagosomes in each cell was 6.0 ± 1.3% and 3.5 ± 1.2% in control and celastrol (1 μM), respectively (Figure 5A). MPP^+^ treatment reduced the percentage of colocalization to 2.3 ± 0.9% as compared to control (Figure 5A). Celastrol cotreatment with MPP^+^ (1 mM) for 4 h enhanced MPP^+^-induced damaged mitochondria co-localization with autophagosomes to 16.7 ± 2.3% (arrows indicate co-localization signals of mitochondria-containing autophagosomes) as compared to MPP^+^ (Figure 5A). Parkin-mediated mitophagy requires p62 to bind with parkin-ubiquitylated mitochondrial substrates and then with LC3-II for further mitochondrial degradation [35]. After cells were treated with MPP^+^ (1 mM) for 4 h to induce mitochondrial damage, mitochondria were isolated from cytosol for mitochondrial protein binding analysis. Western blot results show EF-Tu expressed only in mitochondrial fractions and GAPDH expressed only in cytosol fractions that provided the purity of mitochondrial fraction and cytosol fraction in all groups (Figure 5B). Figure 5B shows that celastrol but not MPP^+^ enhanced mitochondrial ubiquitination in mitochondrial fractions as compared to control. Celastrol cotreatment with MPP^+^ reversed mitochondrial ubiquitination as compared to MPP^+^ (Figure 5B). The protein sequence of p62 contains LC3 binding site for the sequestrated of ubiquitylated mitochondria into autophagosomes for mitophagy [36]. MPP^+^ but not celastrol increased p62 conjugated mitochondria as compared to control, whereas celastrol cotreatment with MPP^+^ enhanced p62-labeled mitochondria as compared to MPP^+^ (Figure 5C). We found that LC3-II was mainly localized to mitochondrial fractions and LC3-I mainly in cytosolic fractions, indicating that mitochondria were sequestrated inside autophagosomes (Figure 5B). Furthermore, MPP^+^ but not celastrol treatment inhibited the sequestration of impaired mitochondria into autophagosomes (LC3-II↓) as compared to control, whereas celastrol co-treatment with MPP^+^ reversed it (Figure 5B). These data suggest that MPP^+^ inhibited and celastrol enhanced mitophagy; celastrol removes impaired mitochondria via activating mitophagy to control mitochondrial quality.

## 4. Discussion

**Figure 6:**
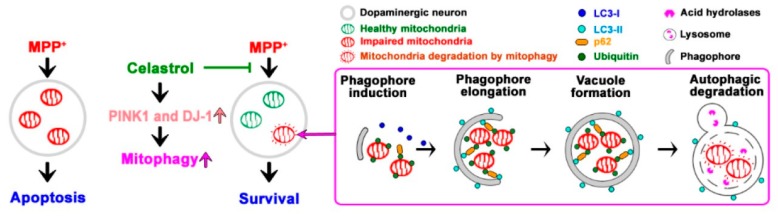
A proposed model showing how celastrol exerts neuroprotection in dopaminergic neurons. MPP^+^ is a mitochondrial complex I inhibitor, which impairs mitochondria function. In this study, MPP^+^ inhibits impaired mitochondrial ubiquitination and further represses mitophagy, which results in dopaminergic neuron apoptosis. Celastrol increases PINK1 and DJ-1 expressions to activate mitophagy for mitochondrial quality control through enhancing (1) autophagosome biogenesis (phagophore induction↑, phagophore elongation↑, and vacuole formation↑), and (2) mitochondrial ubiquitination for p62 recruitment and further LC3-II binding. It results in impaired mitochondria sequestrated by autophagosome for autophagic degradation against MPP^+^-induced dopaminergic neuronal death.

In this study, we used MPP^+^ cell and MPTP mouse models to evaluate celastrol in PD treatment and explore the neuroprotective qualities of celastrol. First, we found that mRNA expressions reveal lower autophagic activity in the substantial nigra from 24 PD patients (*LC3A*↓, *LC3B*↓, and *p62*↑) as compared to 15 healthy controls. In cells, celastrol (1) enhances autophagosome formation (LC3-II↑) and autophagic flux/substrate degradation (p62↓); (2) activates MAPK/p38, MAPK/ERK, MAPK/Akt, and MAPK/JNK signaling pathways; (3) increases autophagosome biogenesis in the processes of phagophore induction (Beclin 1↑, Ambra 1↑, and Vps34↑), phagophore elongation (Atg7↑ and Atg12↑), and vacuole formation (LC3-II↑ and Atg4B↑); (4) enhances mitophagy (PINK1↑, DJ-1↑, and LRRK2↓). In the cellular model of PD, celastrol inhibits MPP^+^-induced neurotoxicity and maintained mitochondrial functions (membrane depolarization↓ and ATP production↑). Also, celastrol maintains mitochondrial quality by engulfing impaired mitochondria into autophagosomes (mitochondria and autophagosomes colocalization↑) for further degradation by mitophagy. In the mouse model of PD, celastrol (1) improves PD motor symptoms (cylinder task↑ and rotarod performance↑), (2) inhibits neurodegeneration (tyrosine hydroxylase↑) in the substantial nigra and striatum, (3) reduces neuronal apoptosis (Bcl-2↑) in the substantial nigra, and (4) enhances mitophagy (PINK1↑ and DJ-1↑) in the striatum. These data suggest that celastrol exerts neuroprotection in PD via mitophagy for the clearance of impaired mitochondria by autophagy degradation and further inhibits dopaminergic neuronal apoptosis. Mitochondrial dysfunction is a prominent phenomenon in the pathogenesis of PD. Removal of damaged mitochondria through mitophagy would therefore greatly impact the disease process. Celastrol is beneficial for the prevention and treatment of PD. Our proposed model showing how celastrol exerts neuroprotection in dopaminergic neurons is depicted in Figure 6.

Mitochondria are essential for cell viability, ATP production, metabolism of reactive oxygen species (ROS), regulation of Ca^2+^ dynamics, and apoptosis [37]. Central nerve system functioning depends heavily on efficient mitochondrial function, given the high energy demands in the brain [38]. In neurons, mitochondria are critical for the maintenance of membrane ion (Na^+^ and Ca^2+^) gradients, neurotransmission, and synaptic plasticity [39]. Neuronal mitochondria are especially susceptible to oxidative stress, as their electron transport chain is very active and therefore generates large amounts of superoxide anion radicals [37]. In this study, we found that celastrol (1) increases protein levels of PINK1 and DJ-1 but represses LRRK2 expression, (2) enhances MAPK/ERK activation, and (3) celastrol maintains mitochondrial membrane potential and ATP production under MPP^+^ treatment. Increasing evidence suggests that PINK1 and DJ-1 are essential for mitochondrial quality control [40]. DJ-1 overexpression induces ERK-dependent mitophagy and protects against rotenone-induced apoptosis [33]. Loss of DJ-1 leads to mitochondrial phenotypes including reduced membrane potential and increased fragmentation [40]. These data suggest that mitophagy is involved in the neuroprotective ability of celastrol.

Mitochondria has an important role in aging-related neurodegenerative disorders such as PD, AD, Huntington’s disease, and amyotrophic lateral sclerosis [38]. PD is a mitochondrial disease of aging [2]. The numbers of mitochondria in a cell and the size of individual mitochondria are malleable and are regulated by the processes of mitochondrial fission and fusion [41]. Fission plays a role in mitochondrial biogenesis and is fundamental to the elimination of dysfunctional mitochondria by mitophagy [35]. Mitophagy is an ongoing process in healthy cells that selectively removes damaged or dysfunctional mitochondria, which could harm the cell by generating excessive amounts of ROS and by releasing pro-apoptotic signals such as cytochrome c [42]. Mitophagy can be stimulated by moderate levels of metabolic and oxidative stress and by inhibition of the mammalian target of rapamycin (mTOR) pathway [41]. The PD-associated protein DJ-1 interacts directly with mitochondria as a redox sensor of mitochondrial stress, while PINK1 plays critical roles in mitophagy and dominant inheritance of mutations in LRRK2 results in abnormal mitochondrial fragmentation [43]. In the present study, we found celastrol regulates DJ-1, PINK1, and LRRK2 expressions in neurons. It provides a new mechanism of celastrol in neuroprotection.

Autophagy contains several steps, including autophagy induction, substrate recognition and selection, phagophore induction and elongation, autophagosome formation, autophagosome-lysosome fusion, and substrate degradation [5]. Autophagy removes aggregated proteins and damaged organelles but excessive or imbalanced induction of autophagy (so-called autophagic stress) contributes to neurodegeneration and cell death [44]. Jezabel et al. (2012) showed that MPP^+^ induces autophagic cell death in nerve growth factor (NGF) differentiated PC12 cells; they found membrane-bound condensed autophagic bodies containing mitochondria accumulated in MPP^+^-treated cells [24]. Suggesting MPP^+^-induced autophagic stress is caused by an impairment of mitochondrial degradation and enhances mitochondrial degradation, which can reduce cell death. In the present study, celastrol activates autophagy in undifferentiated SH-SY5Y against MPP^+^-induced neurotoxicity. We found celastrol enhances autophagosome formation and sequestering impaired mitochondria into autophagosomes for degradation. Indicating celastrol decreases MPP^+^-induced autophagic stress to increase neuronal survival.

PD is a progressive neurodegenerative disorder; there is no effective therapy available to cure PD or significantly inhibit the progression of PD symptoms. Autophagy and mitophagy is a therapeutic approach to PD. Celastrol, a triterpene, is a potent anti-inflammatory and antioxidant that is extracted from the root bark of an ivy-like vine, *Tripterygium wilfordii Hook*, a member of the *Celastraceae* family [20]. In China, this plant has a long history of use in traditional medicine for treating fever and joint pain [20]. In a clinical trial, the root extract of *Tripterygium wilfordii Hook* (2 mg/kg/day) was well tolerated and prolonged remission in patients with Crohn’s disease over 52 weeks [45]. Beside acute pathology, celastrol is used to treat chronic diseases such as cancer, neurodegenerative disease, diabetes, obesity, rheumatoid arthritis, systemic lupus erythematosus, inflammatory bowel diseases, osteoarthritis, and allergy in preclinical studies [46]. Celastrol is of great interest, as its low toxicity makes it easy to test in clinical trials [20]. Since PD is characterized by abnormal protein aggregation and impaired mitochondria, using celastrol to enhance autophagy and mitophagy by removing aggregated protein and dysfunctional mitochondria could be a useful therapeutic strategy in PD prevention and treatment.

## 5. Conclusions

PD is a neurodegenerative disease. No treatment can stop or slow the progression of PD. Autophagy is neuroprotective through removing damaged mitochondria and abnormal protein aggregations in neurons. In the present study, celastrol exerts neuroprotection in PD by activating mitophagy to degrade impaired mitochondria and further inhibit dopaminergic neuronal apoptosis. Celastrol may help to prevent and treat PD.

## Figures and Tables

**Table 1:** Primer and probe sequences of real-time quantitative PCR.

Gene	Forward primer	Reverse primer	Probe
*Beclin1*	GGATGGTGTCTCTCGCAGTA	TTGGCACTTTCTGTGGACAT	CCTGGAGC
*Abmra1*	CCTCTCCTCCACAATTTCCTG	GATGGAAGGGCTCGGTCT	GGACAGCA
*Atg7*	TGGCTGCTACTTCTGCAATG	CAAGGTCCGGTCTCTGGTT	CTGGGGCC
*Atg12*	TTGTGGCCTCAGAACAGTTG	CCAAAACACTCATAGAGAGTTCCAA	CTTCAGCC
*LC3A*	CATGAGCGAGTTGGTCAAGA	CACCATGCTGTGCTGGTT	TCCTGCTG
*Atg4B*	ATTGGTGCCAGCAAGTCAA	GCAGGCCAGATGTGAAGG	TGGTGGAG
*DJ* *-1*	GATGTCATGAGGCGAGCTG	TGACCACATCACGGCTACAC	CCTGGAGC
*PINK1*	GCCATCAAGATGATGTGGAAC	GACCAGCTCCTGGCTCATT	CTGGAGGA
*β-actin*	ATTGGCAATGAGCGGTTC	CGTGGATGCCACAGGACT	CTTCCAGC
